# Mpox: The alarm went off. Have we gone back to sleep?

**DOI:** 10.1371/journal.pntd.0011871

**Published:** 2024-01-18

**Authors:** Piero Olliaro, Josephine Bourner, Yap Boum II, Emmanuel Nakouné, Elise Pesonel, Amanda Rojek, Yazdan Yazdanpanah, François-Xavier Lescure, Alexandra Calmy, Beatriz Grinsztejn, Peter Horby, Laura Merson, Jake Dunning

**Affiliations:** 1 International Severe Acute Respiratory and emerging Infection Consortium (ISARIC), Pandemic Sciences Institute, University of Oxford, Oxford, United Kingdom; 2 Institut Pasteur de Bangui, Bangui, Central African Republic; 3 ANRS: Maladies Infectieuses Emergentes, Paris, France; 4 Infectious diseases department, Bichat hospital, APHP, Paris Cité University, IAME UMRS 1137 Inserm, Paris, France; 5 Hôpitaux Universitaires de Genève, Geneva, Switzerland; 6 Instituto Nacional de Infectologia Evandro Chagas, FIOCRUZ, Rio de Janeiro, Brazil; NIAID Integrated Research Facility, UNITED STATES

Right at the outset of an outbreak that was later declared a Public Health Emergency of International Concern (PHEIC), we hoped there would be a wakeup call for the international community to pay attention to mpox [1]. It did, but not quite enough. Despite some 91,000 cases of clade IIb mpox virus registered worldwide to-date [2], little progress has been made into gathering essential evidence as to what works or does not in preventing and treating mpox, with stark inequalities in access to therapy and vaccination between high-income and low- and middle-income countries, including the African countries with recurring outbreaks of clade I and clade IIa mpox virus [3].

While the PHEIC has been successfully curbed, we see again the challenges of mounting an effective and timely clinical research response within the timeframe of relatively short-lived, geographically dispersed outbreaks. The search for safe and effective treatments is a clear example of this. Ahead of the 2022 clade IIb outbreak there were 2 main candidate therapeutics: tecovirimat and brincidofovir [4]. They have shown activity in vitro and in animal models, but their clinical effectiveness in humans is uncertain. Tecovirimat is currently registered as a treatment for mpox in the European Union (EU) and the United Kindgom (UK), based on “exceptional circumstances.” In the United States (US), tecovirimat is approved only for smallpox, under the “animal rule” exception for treatments for which human efficacy studies are not ethical or feasible and can be obtained for mpox through the Centers for Diseases Control and Prevention’s Expanded Access Investigational New Drug “compassionate use” protocol [5,6]. No similar mechanisms appear to exist at present for brincidofovir. As for clade I endemic countries, tecovirimat can be accessed under expanded access programme [7] in the Central African Republic (CAR) [8] where the treatment is not registered. Additionally, cidofovir is also being used off-label.

**Fig 1:**
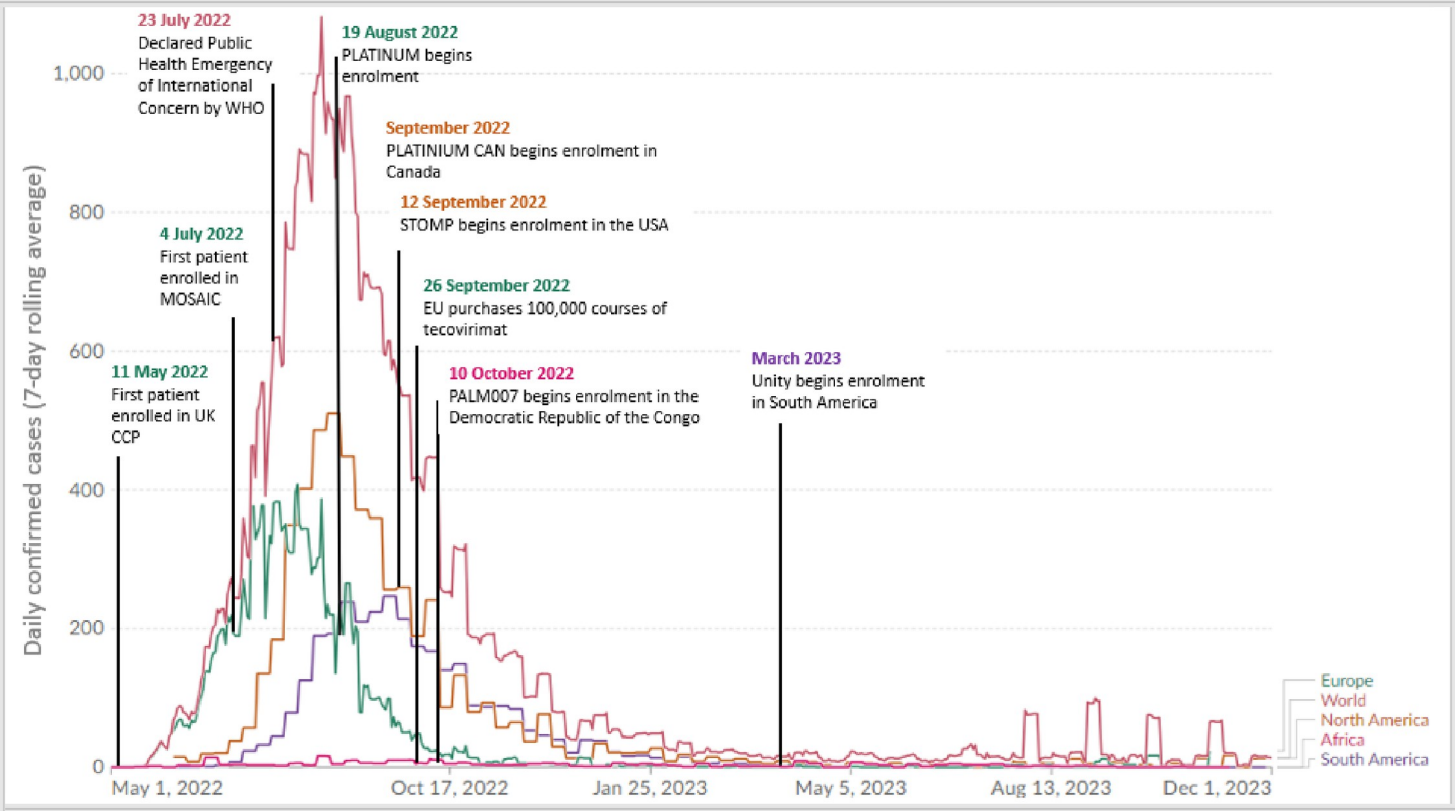
Timeline of the events during the 2022 multicountry mpox outbreak and daily confirmed cases (7-day rolling average). Image credit: Our World in Data: mpox [9].

The current research landscape for mpox therapeutics—made up of both interventional and observational studies as well as Expanded Use Protocols (EAP)—is summarised in **Table 1**. While the situation is evolving, it is clear that, bar a major rebound of cases, this has been a missed opportunity for gathering evidence on the management of clade IIb cases (**Fig 1**).

**Table 1:** Summary of mpox research studies and programmes taking place as of December 2023.

Study type	Study title	Sponsor	Location	Clade primarily studied	Target sample size	Status	Trial registration ID
RCT	PLATINUM-UK	University of Oxford	United Kingdom	Clade IIb	450	Stopped recruiting	ISRCTN17461766
RCT	PLATINUM-CAN	McGill University	Canada	Clade IIb	120	Not yet recruiting	NCT05534165
RCT	STOMP	NIAID/DAIDS	Multiple countries	Clade IIb	530	Actively recruiting	NCT05534984
RCT	UNITY	Hospital University Geneva (HUG)	Brazil, Argentina, Switzerland	Clade IIb	150	Actively recruiting	NCT05597735
RCT	EPOXI	UMC Utrecht ECRAID	Multiple countries (Europe)	Clade IIb	644	In set-up	NK
RCT	MOSA	PANTHER	Multiple countries (Africa)	Clade I and IIa	500	In set-up	NK
RCT	PALM007	NIAID	Democratic Republic of Congo	Clade I	450	Actively recruiting	NCT05559099
EAP	-	US Army Medical Research and Development Command	United States	Clade IIa	NA	Actively recruiting	NCT02080767
EAP	Tecovirimat for mpox in CAR	University of Oxford	Central African Republic	Clade I	NA	Actively recruiting	ISRCTN43307947
MEURI	-	WHO	Global	All clades	NA	Actively recruiting	NA
Observational/LICT	MOSAIC	University of Oxford	Multiple countries (Europe)	Clade IIb	NA	Stopped recruiting	2022-501132-42-00
Observational	UK CCP	University of Oxford	United Kingdom	All clades	NA	Actively recruiting (stopped recruiting clade IIb)	NA
Observational	NETPOX	Hospital Israelita Albert Einstein	Brazil	Clade IIb	80	In set-up	NCT05784038

CAR, Central African Republic; CTIS, Clinical Trials Information System; EAP, Expanded Use Protocol; EMA, European Medicine Agency; EU, European Union; LICT, Low Interventional Clinical Trial; PHEIC, Public Health Emergency of International Concern; UK, United Kingdom; US, United States.

While recruitment to one study, MOSAIC, started in July 2022, no more than 2 months after the outbreak commenced, most active trials began recruiting after the peak of the epidemic had passed and cases were already in a rapid decline (**Fig 1**), and as of October 2023, the rest are not recruiting. The EAP taking place in the US has to-date included almost 7,000 patients; while so far longitudinal data are publicly available from only on a minority of treated patients [9], it is hoped that more data will be released soon [10].

For clade I, the ongoing randomised controlled trial (RCT)—PALM 007—in the Democratic Republic of the Congo will hopefully provide a definitive answer to the effectiveness of tecovirimat for this clade. Meanwhile, the EAP taking place in CAR has treated so far 25 confirmed cases of mpox and collected data on clinical outcomes over at least 28 days.

While study protocols for clade IIb could be developed by adapting available clade I protocols, hurdles were faced in mounting a timely clinical research response to the clade IIb outbreaks, the reasons for which are both systemic and disease specific.

The systemic issues are in the inordinate amount of time that it takes after a protocol is developed to get through regulatory processes before enrolment can start. The lessons we learned from COVID-19 have not been used to make the system more efficient. The MOSAIC study, for example, which collects data on clinical and virological outcomes of patients with laboratory-confirmed mpox, has been the victim of a convoluted regulatory landscape that led to the study being treated as both an observational study in the UK and Switzerland and a Low Interventional Clinical Trial (LICT) in the EU—the latter requiring submission and review via the European Medicine Agency’s (EMA) Clinical Trials Information System (CTIS) platform and compliance with the EU Clinical Trial Regulation (despite no treatment being administered or altered under the MOSAIC protocol). Despite the declaration of a PHEIC, the median approval time for the study in the EU was 46.5 days, compared to 14 days in the UK and 20 days in Switzerland [11]. As a result, some of the prospective participating countries “missed the boat” as they could not start recruitment due to delays in contract negotiation. The true burden of these delays becomes evident when these prospective studies are compared with one of the success stories of mpox—the rapid generation of clinical characterisation and outcome data from retrospective case-data (such as SHARE-NET). These collaborations were often quick to produce novel clinical insights (such as cohorts of women [12] and people living with HIV/AIDS [13], or descriptions of re-infection following vaccination [13]). They demonstrate a strong desire from clinical researchers to commit to international collaboration and rapid evidence generation—and a clear dividend of doing so when not encumbered by regulatory barriers. A key frustration is that there is no to negligible difference in the level of risk to patients between these case series and observational cohorts such as MOSAIC.

More generally, due to the moving nature of clade IIb outbreaks (see Fig 1), current studies may not actually cover all areas of ongoing transmission. The present clinical research ecosystem is not designed to favour international collaborations establishing pre-positioned protocols and “dormant” studies which could be activated where and when cases occur.

There are also complications that are more disease specific. Differences in transmission routes [14], virology [15], and other unknown factors cause the variation in clinical presentation between clade IIb (also referred to as clade III) [16] and the known clade IIa and clade I patterns. Decisions on methodological issues lacked the benefit of consistently recorded clinical characterisation data for clade I (and the early cases for clade IIb), leading to uncertainty in trial protocols that could have impacts on sample size and efficiency. While most interventional and observational studies now share broadly consistent criteria and outcomes, uncertainties remain about study endpoints [17] and about reliability and agreement among clinicians in clade IIb lesions assessment [18].

These considerations are not unique to mpox. Most high-consequence pathogens cause outbreaks that are short-lived, geographically confined or sometimes dispersed, often occurring in difficult environments in low-resource countries where they affect marginalised populations. These diseases are neglected because of “market failure,” as they do not represent an appealing “business case” for profit-driven pharmaceutical companies. Yet, investing in collaborative research into these diseases would be crucial both to ensure equitable access to healthcare for marginalised populations, and also to be better prepared for potential further onward transmission and spread to wider geographical areas, including high-resource countries.

While it will never be possible to be entirely prepared for new outbreaks, we know the essential ingredients of an effective clinical research response.

First, a coherent strategy making the best use of observational studies—to give information on risk factors, clinical characterisation and expected outcomes—together with interventional clinical trials—to generate robust safety and efficacy data on vaccines and treatments. A case in point in the recent mpox outbreak being the speed at which cohort studies and case series were able to generate a substantial amount of data characterizing hundreds of patients across a diverse population [12,13,19,20].

Second, international collaborations to reach larger numbers of participants and greater geographic diversity, leading to results that are more generalizable, based on agreed-upon standardized or, at least, compatible, methodologies. Such has been the case for mpox, although the ongoing clade IIa studies may not reach the planned sample sizes. To offset these shortcomings, at least in part, a meta-analysis of individual patient data that is currently under discussion among investigators of placebo-controlled trials of tecovirimat. Creative collaborations and research framework are essential to provide rapid answers to health emergencies.

Third, a regulatory and political environment enabling processes and systems to support research, from prompt, adequate funding to rapid reviews of clinical trial applications so that research can take place where and when cases occur and have a chance to reach the planned sample size and generate robust evidence.

Fourth, investing into strengthening clinical research capacity in outbreak-prone areas beyond frontiers is not only an essential integral part of the preparedness strategy, but also a way to help low-resource countries build the agency to deal with locally relevant priority health issues.

Lastly, upfront commitments to equitable, affordable access to medical interventions found to be safe and effective in studies [17].

Lessons should have been learnt from COVID-19, but they were not acted upon for mpox. All actors in the research community—from individual research teams to regulators, to political and public health decision-makers to drug developers to legal departments at research sites—must step up and address pressing issues to provide an enabling environment for essential clinical research in outbreak response.
